# Mesorectal motion evaluation in rectal cancer MR-guided radiotherapy: an exploratory study to quantify treatment margins

**DOI:** 10.1186/s13014-022-02193-1

**Published:** 2023-01-06

**Authors:** Luca Boldrini, Giuditta Chiloiro, Davide Cusumano, Angela Romano, Lorenzo Placidi, Gabriele Turco, Marco Valerio Antonelli, Matteo Nardini, Matteo Galetto, Luca Indovina, Maria Antonietta Gambacorta

**Affiliations:** 1 Fondazione Policlinico Universitario “A. Gemelli” IRCCS, Largo Agostino Gemelli 8, 00168 Rome, Italy; 2 Mater Olbia Hospital, Strada Statale Orientale Sarda 125, Olbia, SS Italy; 3 Università Cattolica del Sacro Cuore, Largo Francesco Vito 1, 00168 Rome, Italy

**Keywords:** Magnetic resonance guided radiation therapy, Rectal cancer, Mesorectal motion, Treatment personalization

## Abstract

**Background:**

Mesorectal motion (MM) is a source of uncertainty during neoadjuvant chemoradiotherapy (nCRT) delivery for locally advanced rectal cancer (LARC). Previously published experiences using cone-beam computed tomography imaging have already described significant movement. Aim of this analysis is to assess inter-fraction MM using the higher tissue contrast provided by hybrid magnetic resonance imaging (MRI) in LARC patients (pts) treated with MRI guided radiation therapy (MRgRT).

**Methods:**

The total mesorectum, its superior (Msup), middle (Mmid) and lower (Mlow) regions were contoured on the positioning MRIs acquired on simulation day and on each treatment day. Six PTVs were obtained adding 0.5, 0.7, 1, 1.3, 1.5 and 2 cm margin to the whole mesorectum, starting from the simulation MRI. Margins including 95% of the mesorectal structures during whole treatment in 95% of patients (pts) were considered adequate.

**Results:**

A total number of 312 fractions of 12 consecutive pts was retrospectively analyzed. The different mesorectum regions show specific motion variability. In particular, Msup shows larger variability in left, right and anterior directions, while the Mlow in caudal and posterior ones. The anterior margin is significantly larger in the Msup than in the other regions.

**Conclusion:**

Different mesorectal regions move differently throughout the radiotherapy treatment, with the largest MM in the Msup anterior direction. Asymmetrical margins are recommended.

## Background

Radiotherapy (RT) techniques for locally advanced rectal cancer (LARC) treatment have evolved in recent years. The transition from 3D conformational RT (3D-CRT) to intensity modulated radiation therapy (IMRT) and volumetric arc therapy (VMAT) has led to more precise dose conformation and consequent more efficient organs at risk (OARs) sparing. With the use of the modern technologies implementing image guided radiotherapy (IGRT) is now possible to reduce the dose to OARs by creating steep dose gradients between tumor and surrounding healthy tissues [1]. In this perspective, it is possible to implement dose escalation protocols, as dose intensification to the tumor has been shown to increase the probability of pathological complete response (pCR) in an organ preserving strategy [2].

The aim of IGRT is to reduce the delivery uncertainties related to patient positioning and anatomy variation, considering the possible anatomical changes during RT treatment. These uncertainties can be mitigated using bowel and bladder preparation protocols, appropriate immobilization devices and IGRT solutions. The modern IGRT methods include electronic portal imaging devices (EPIDs), cone beam computed tomography (CBCT), kilovoltage or megavoltage CT scans, tracking of fiducials. However, radiological imaging approaches present critical issues in visualising soft-tissues, limiting the evaluations on patients’ set-up exclusively on the bony and making critical the correct delineations of the therapy volumes anatomy [3].

In case of patients affected by LARC, the mesorectum is part of the Clinical Target Volume (CTV), although its identification results to be challenging due to the poor visualisation with traditional IGRT on-board solutions [4].

Furthermore, the mesorectum is a very mobile structure, as described in previous reports, especially along the antero-posterior axis of its antero-superior sections [5–7].

Mesorectum motion (MM) has already been analyzed in several reports, investigating the appropriate margins to be added from CTV to planning target volume (PTV) which currently range from 1 to 1.5 cm [8–11], however most of these reports used CT or CBCT-based imaging and with limited observations during the treatment, especially in the long course treatment.

The MM and anatomical modifications may therefore cause dose delivery uncertainties that may play a significant role not only in terms of dose intensification, especially in the perspective of a conservative surgical approach [12].

In recent years, more conservative approaches such as local excision or watch and wait have been adopted for patients with favourable prognosis who have achieved a complete or major clinical response (CR) after neoadjuvant chemoradiotherapy (nCRT) [13, 14]. Although such approaches do not represent the standard of treatment, in this perspective a clear modelling of the MM is even more important in order to apply adequate margins to treatment volumes.

The recent introduction of hybrid systems coupling on-board Magnetic Resonance (MR) scanner and linear accelerator has led to the clinical introduction of Magnetic Resonance guided Radiotherapy (MRgRT) and the availability of 3D daily MR image for contouring and patients positioning [15].

Aim of this study was to analyze the MM on different LARC patients, starting from the 3D MR images acquired each day of therapy, with the aim of verifying the validity of the PTV margins proposed in the previous studies and to suggest customised treatment margins.

## Methods

### Patient data and treatment details

We retrospectively analysed data from consecutive LARC patients undergoing nCRT and treated on 0.35T MRgRT unit (MRIdian, ViewRay Inc, Mountain View, CA, USA) from March 2017 to April 2018. All patients underwent medical examination, staging with pelvic MR, chest and abdomen CT scan and endoscopic procedure with biopsies. The indication for nCRT treatment was agreed in the context of multidisciplinary institutional tumor board, consisting of radiation oncologists, medical oncologists, radiologists, pathologists and surgeons.

Patients aged higher than 18 years, ECOG 0–1, without contraindications to perform MR examinations were included. Specific informed consent for MRgRT was also obtained. Neoadjuvant CRT treatment was prescribed according to a Simultaneous Integrated Boost 2 (SIB2) delivery protocol, according to our institutional guidelines. In particular, 5500 cGy in fractions of 220 cGy were prescribed to CTV 1, consisting of the site of primary disease (gross tumor volume - GTV) plus the corresponding mesorectum with 5 mm isotropic margin, while 4500 cGy in fractions of 180 cGy, consisting of mesorectum in toto and selected lymphatic drainage stations according to disease stage with 5 mm isotropic margin, to PTV2 [4]. In the case of extramesorectal lymph nodes involvement, a boost on GTV_N was performed [16].

Concomitant chemotherapy with chronomodulate Capecitabine (1650 mg/mq)/5-Fluorouracil (5-FU) c.i. or an intensification schedule with Capecitabine (1300 mg/mq) plus Oxaliplatin (60 mg/mq) was prescribed, in relation to clinical stage and general conditions of the single patient [17].

Patients were immobilised in the supine position, using the Fluxboard device (Fluxboard™, MacroMedics, The Netherlands) in an appropriate, personalised, and comfortable configuration [18]. All patients performed bladder preparation by drinking 500 cc of water before the simulation and before each therapy session; no rectal or bowel preparation was performed.

At the time of the simulation, a 175-seconds MR true fast imaging (TRUFI) was acquired with subsequent T2*/T1 image contrast in free breathing (FB) mode. After approximately 20 min, a simulation CT was acquired in the same treatment position to acquire electron densities for planning purposes.

Furthermore, a 175-seconds positioning MR scan was acquired prior to all the 25 therapy fractions [18].

### Delineation details

A radiation oncologist retrospectively contoured mesorectum, bladder, rectum and bowel bag of all the treatment fractions for all the analysed patients, including simulation MRI. This resulted in the different CTVs, named CTV_sim, CTV_Fx1, CTV_Fx2…CTV_Fx25, as well as for bladder, rectum, and bowel bag, following the same nomenclature, for each patient.

Furthermore, GTVs at the time of simulation, and at fractions 5, 10, 15, 20 and 25 (GTV_sim, GTV_Fx5, GTV_Fx10, GTV_Fx15, GTV_Fx20, GTV_Fx25) were delineated for all the patients, to assess whether GTV variations in terms of location and volume may have an impact on MM.

Contouring was performed on the high resolution 175-s TRUFI MR scan according to the international guidelines using the MRIdian workstation [4].

In particular, the mesorectum was delineated from the bifurcation of the inferior mesenteric artery (IMA) into sacral artery (SA) and superior rectal artery (SRA) and down to the insertion of the elevator ani muscle into the external sphincter muscles, when the mesorectal fat around the rectum disappears.

The mesorectum is surrounded by the mesorectal fascia, which is delimited posteriorly by the anterior surface of the sacrum and in the mid-lower pelvis anteriorly by the posterior border of the anterior pelvic organs (prostate, seminal vesicles, bladder and penis bulb in men and vagina and uterus in women [4]. The total mesorectum, the superior mesorectum (Msup), the middle mesorectum (Mmid) and the lower mesorectum (Mlow) were considered for this analysis.

**Fig. 1:**
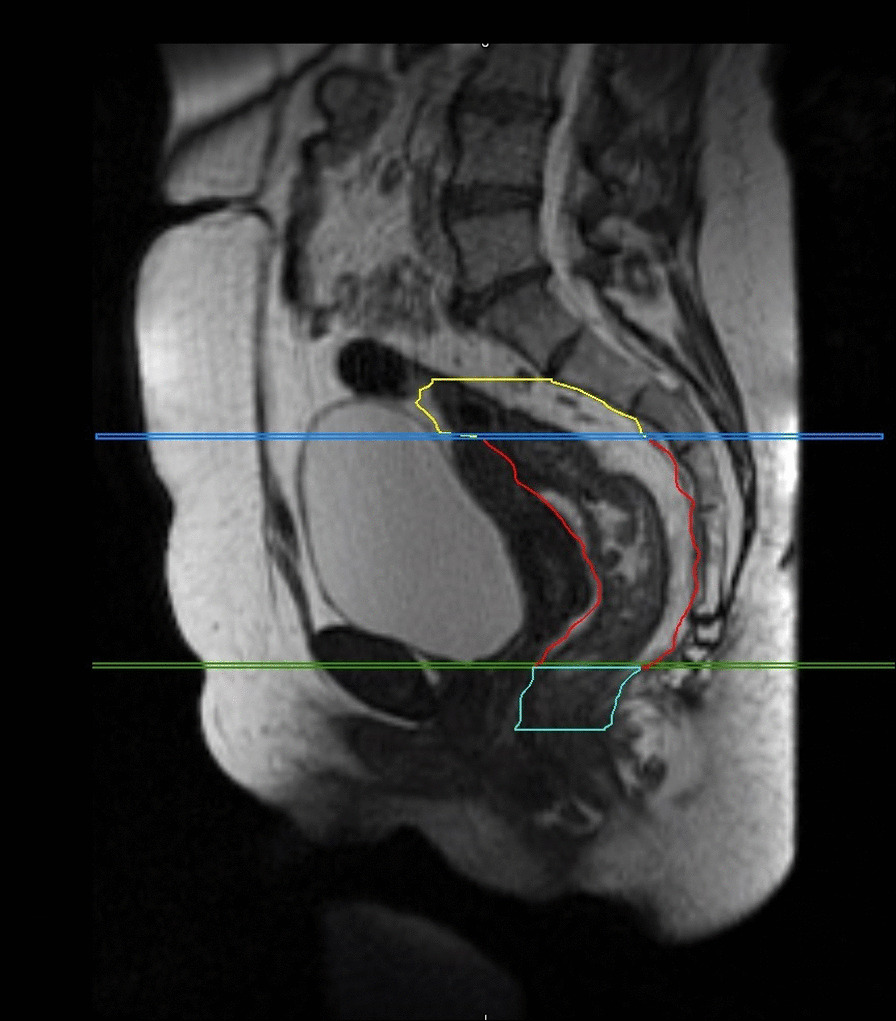
Sagittal scan of the mesorectum subdivision into three sections obtained through two axial planes. The superior mesorectum (yellow) is separated from the middle mesorectum (red) by the axial plane passing through the sigmoid-rectal junction (blue). The latter is then divided from the lower mesorectum (light blue) by a plane passing through the recto-uterine pouch for female and the recto-vesical pouch for male patients (green)

The Msup, Mmid and Mlow were obtained by defining two axial planes as reported in Fig. 1.

Msup is extended from the cranial limit of the mesorectum (bifurcation of the IMA into SA and superior rectal artery SRA) until a plane passing through the sigmoid-rectal junction, when the mesorectal fascia anteriorly is most visible.

Mmid is defined, cranially, from the caudal limit of the upper mesorectum, to a plane passing through the recto-uterine pouch for female and the recto-vesical pouch for male.

Finally, Mlow is defined as the section below the recto-uterine pouch for female and the recto-vescical pouch for male up to the most caudal limit when the fat disappears.

All segmentations were then reviewed by a second radiation oncologist with large experience in rectal cancer for quality assurance purposes.

### Motion analysis

Organ motion was considered for both mesorectum and OARs. All 175-s TRUFI MR scans with all contour sets were transferred to MIM^®^ software (MIM Maestro, version 7.1.2, Maastricht, Belgium) to perform motion analyses. The simulation MR scan and its contours were considered as reference image.

For each patient, all the daily MR images were rigidly registered to the reference image, basing on bony anatomy. The contour sets of each fraction, from 1 to 25, including the CTV and the corresponding OARs, were then transferred to the reference simulation MR.

For each patient, 6 PTV volumes were obtained by adding 0.5 cm, 0.7 cm, 1 cm, 1.3 cm, 1.5 cm, and 2 cm margins to the CTV_sim respectively. These volumes were named progressively PTV_sim, PTV_0.7, PTV_1…, PTV_2.

The overlap of the CTV of all fractions and each of the 6 PTV volumes obtained as described above was evaluated for all patients.

The inter-fractional MM was studied counting the number of fractions where the whole mesorectum was included in the different PTVs margins.

Such analysis was performed separately for the Msup, Mmid and Mlow, to investigate if different mesorectum parts would show motion variability.

The minimum margins that included 95% of the mesorectal structures during the whole treatment in at least the 90% of patients were considered adequate for MRgRT treatments. Considering that the study was conducted on 12 patients treated with 25 fractions, the optimal margins were those able to cover 24/25 fractions (96%) in 11/12 patients (91.6%).

### Correlation analysis

The correlation between mesorectum and surrounding OARs (i.e. bladder, bowel and rectum) was studied patient-by-patient, with the aim of identifying the possible causes of inter-fraction variability of mesorectum.

Variations in mesorectal geometric center during RT treatment were correlated with the change in GTV volume using Pearson Correlation Coefficient (PCC).

Parameters showing PCC higher than 0.7 were considered as highly correlated.

For each couple, the percentage of patients showing high correlation was calculated, in order to investigate if some correlation was common among different patients.

## Results

A total number of 312 0.35T MR sequences obtained during the simulation and during the nCRT treatment for LARC of 12 consecutive patients, treated from March 2017 to April 2018, were included in our analysis.

Patient clinical characteristics are shown in Table 1.

**Table 1:** Patients’ clinical characteristics

Characteristics	N	%
Median age (range)	70 (41–87)	
*Gender*		
Male	10	83.3
Female	2	16.7
*T stage*		
T3	7	58.3
T4a	3	25
T4b	2	16.7
*N stage*		
N+	11	91.7
N0	1	8.3
*Distance from the anal margin (cm)* [19]		
0–5	5	41.7
5–10	4	33.3
> 10	3	25

**Fig. 2:**
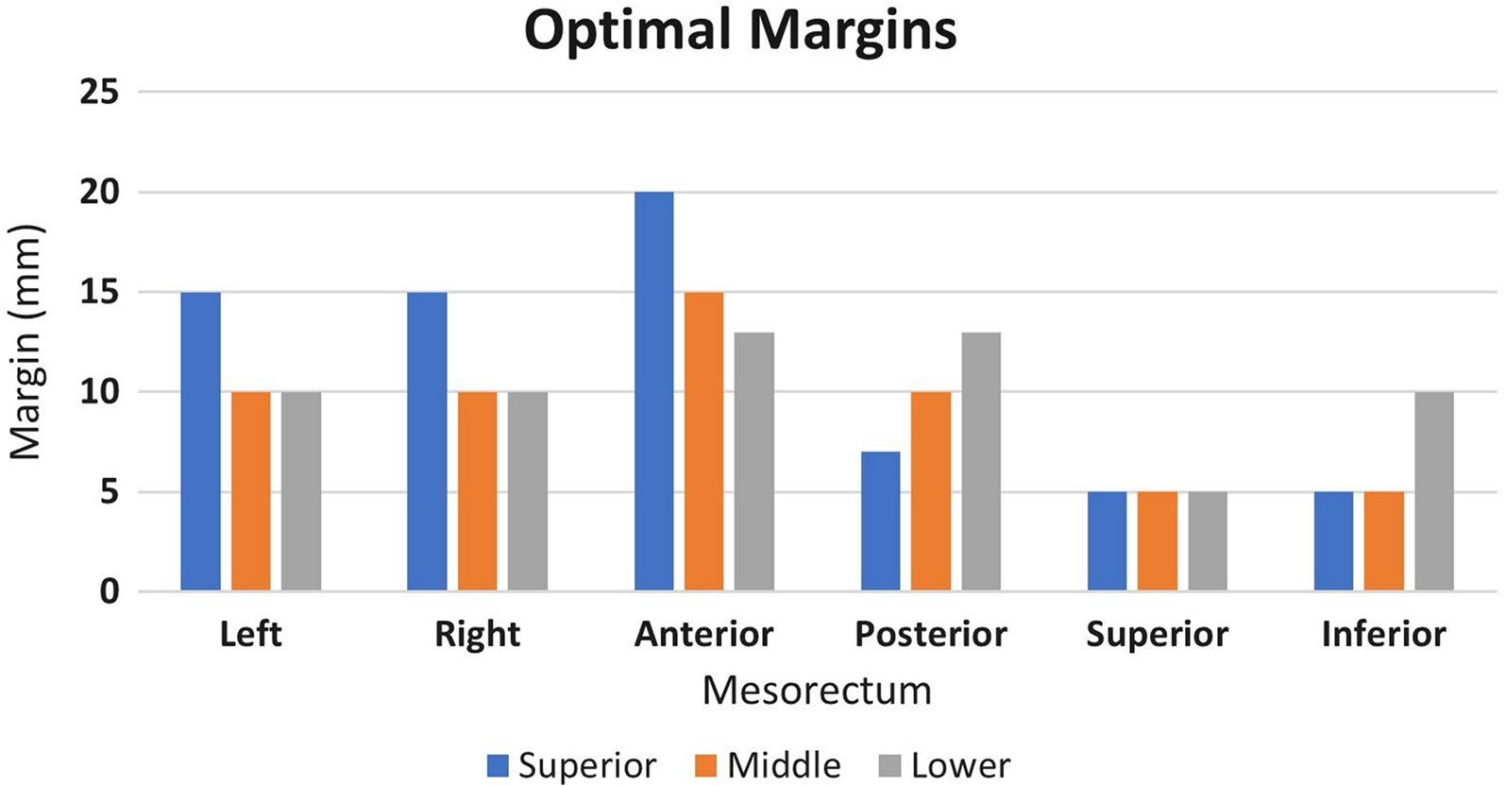
Optimal PTV margins for the different mesorectal sections

The Fig. 2 summarizes the optimal margins obtained for the three mesorectum regions based on the analysis performed on the 12 patients.

Figure 2 shows that different mesorectum regions have different motion variability: Msup shows larger variability in left, right and anterior directions, while the Mlow shows larger variability in caudal and posterior ones. The anterior margin is significantly larger in the Msup (2 cm) than in the Mmid and Mlow, where 1.5 and 1.2 cm are recommended, respectively.

The ideal PTV margins, taking into account the motion of the total and the three mesorectum sections, are summarised in Table 2.

**Table 2:** Suggested PTV margins (in cm) for the total mesorectum and for the 3 mesorectum sections

	Total mesorectum	Msup	Mmid	Mlow
Left	1.5	1.5	1	1
Right	1.5	1.5	1	1
Anterior	2	2	1.5	1.2
Posterior	1.3	0.7	1	1.3
Up	0.5	0.5	0.5	0.5
Down	1	0.5	0.5	1

*Msup*, superior mesorectum; *Mmid*, middle mesorectum; *Mlow*, lower mesorectum

**Fig. 3:**
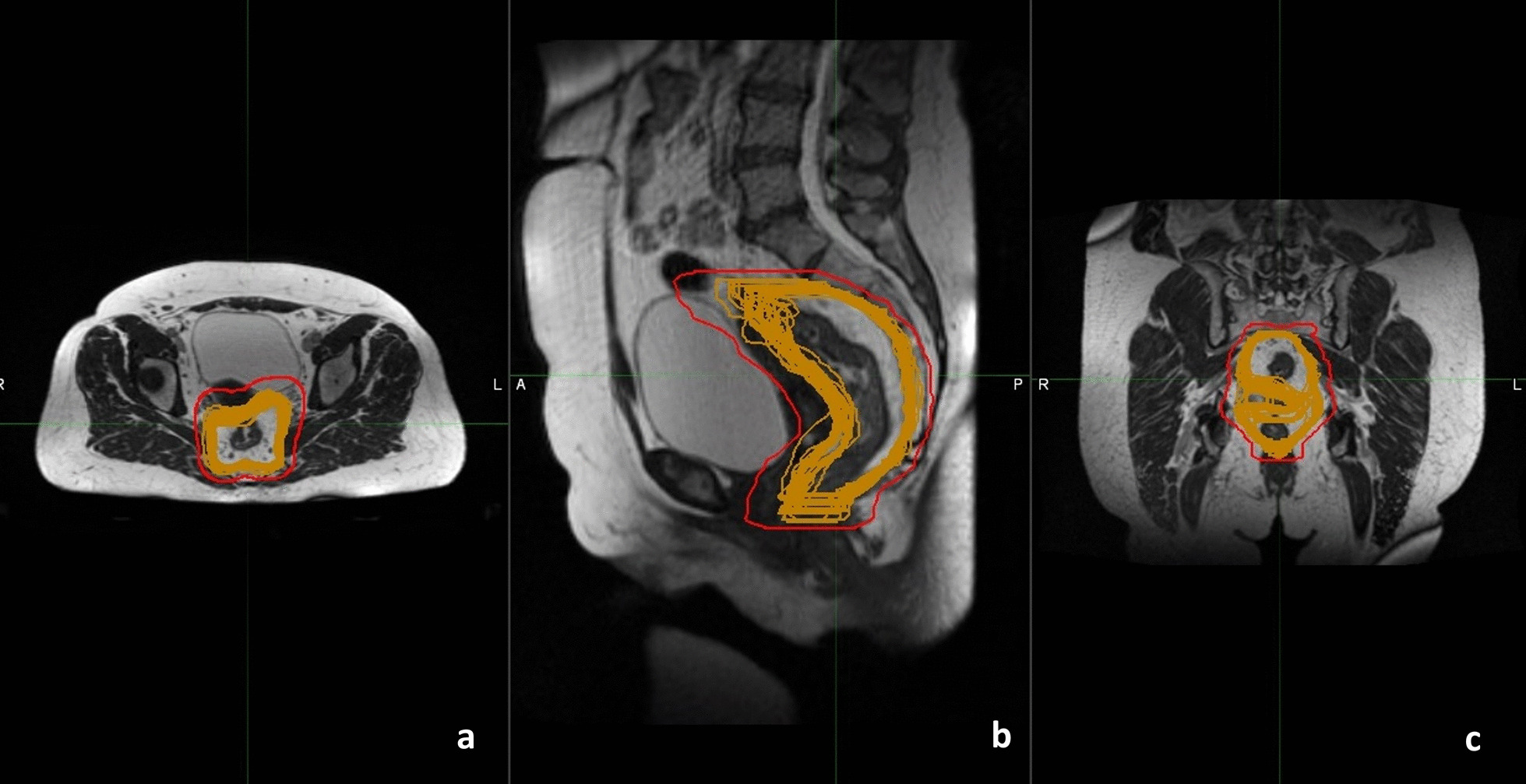
MR scans in the axial (**a**), sagittal (**b**) and coronal (**c**) plans of the mesorectal structures of each treatment fraction rigidly co-registered on the planning fraction (all in brown). The PTV obtained taking into account the inter-fraction motion by applying asymmetric margins to the mesorectum in planning fraction is shown in red

Figure 3 shows the planning MR scans in the three axial, sagittal and coronal plans of a patient and the mesorectal segmentations obtained throughout the treatment course. Following the results obtained in our study, asymmetrical margins (more specifically: 0.5 cm in the cranial direction, 1 cm caudal, 1.3 cm posteriorly, 2 cm anteriorly and 1.5 cm in both anterior and posterior directions) were applied from the mesorectal planning structure to obtain the PTV that takes into account inter-fraction MM.

The results in terms of PCC for the correlation analysis between the volumetric variation of Msup, Mmid and Mlow and the volumetric variations of bladder, rectum and bowel are reported in Table 3. The only significant correlation was observed between bladder and Mmid volume, observed in 4/12 patients.

**Table 3:** PCC results for the correlation analysis between the volumetric change in the superior, middle and lower mesorectum and the volumetric changes in the bladder, rectum and bowel

	Msup			Mmid			Mlow		
Pt	Bowel	Rectum	Bladder	Bowel	Rectum	Bladder	Bowel	Rectum	Bladder
1	0.30	0.27	0.53	0.19	− 0.40	− 0.01	0.14	0.16	0.42
2	0.02	− 0.14	− 0.10	− 0.09	− 0.43	0.45	0.10	− 0.32	0.36
3	0.11	0.52	0.37	0.37	− 0.19	**0.78**	0.00	− 0.53	0.39
4	− 0.23	**0.79**	0.46	0.11	− 0.14	**0.81**	− 0.06	− 0.25	0.18
5	− 0.17	0.52	0.28	0.07	0.00	**0.76**	− 0.05	− 0.05	0.45
6	0.42	0.38	0.57	− 0.27	0.09	0.41	− 0.31	− 0.22	0.06
7	0.00	0.14	0.03	− 0.03	0.07	0.33	− 0.11	− 0.39	0.33
8	− 0.24	0.55	0.33	0.13	0.09	0.28	0.02	− 0.29	− 0.30
9	− 0.01	0.31	0.00	− 0.07	0.13	− 0.17	0.05	− 0.25	0.19
10	0.27	− 0.15	0.09	0.07	**− 0.77**	0.20	0.31	− 0.49	0.32
11	0.62	**0.77**	0.71	0.45	0.51	0.73	− 0.38	− 0.38	− 0.15
12	− 0.64	0.32	0.45	− 0.51	0.30	0.00	0.17	0.48	− 0.14
Significant correlation frequency	0	2	1	0	1	4	0	0	0

Significant values are shown in bold

*Pt*, patient; *Msup*, superior mesorectum; *Mmid*, middle mesorectum; *Mlow*, lower mesorectum. The last row of Table 3 reports the number of cases where a significant correlation was observed: the only significant correlation was observed between bladder and medium mesorectum volume (4/12 patients)

No correlation was found between MM and mesorectal volume in the majority of patients, except for the anterior MM, that was correlated with the mesorectal volume reduction during treatment.

Furthermore, no significant correlation was observed between MM and GTV volume variation.

## Discussion

The present study provides information on inter-fraction MM using daily MR-Linac data obtained during long course nCRT for LARC. Both the rectum and mesorectum are anatomically mobile structures, characterised by positional and volume changes due to peristalsis or organ filling. Guidelines and previous studies suggested large and variable margins to be considered in contouring accurately to account for MM [11, 20]. Sometimes, to avoid the risk of geographic missing resulting in potential local recurrence, wide margins are suggested which may include the posterior bladder wall and bowel loops, potentially resulting in increased urinary and bowel toxicity.

Adequate total mesorectal excision (TME) leads to a reduction in local recurrence and thus an increase in overall survival. It has to be considered that not all TMEs are adequate, especially for the lower mesorectal sections [21–23].

Previous studies conducted on cone beam CT of patients treated in both prone and supine positions have shown increased mesorectum displacement in the cranio-caudal and antero-posterior directions, suggesting the possible application of lower CTV-PTV margins in the latero-lateral direction [5, 24, 25]. Chong et al. [9] analysed the motion of rectum during nCRT and found larger variations in both rectal diameter and anterior- ateral wall movement of the mid and upper rectum compared to the low rectum. Nuyttens et al. [11] reported similar findings, analysing the rectal motion during adjuvant long course RT using weekly CT scans. Although the authors also showed greater rectal wall mobility in the mid and upper rectum, the results are not directly comparable for the post-operative anatomical setting.

Accordingly, we conducted a sub-analysis considering the different mesorectal sections and the required CTV-PTV margins appropriate for each one of them. Our results confirmed those reported by previous studies, showing a significantly larger anterior margin mobility in the superior mesorectal section (2 cm) compared to the middle and lower, where 1.5 cm and 1.2 cm margins are required, respectively. As concluded also in previous studies, we recommend the use of an anisotropic margin.

Among the potential factors that could influence the mobility of the different mesorectal sections, the volume variation of bladder, bowel and rectum itself were evaluated.

This variation appears to have no impact on MM, as also observed by other authors [6, 9]. In our case, it could be due to the daily bladder preparation and nutritional advices given to patients to avoid faecal stasis and the occurrence of gas in the rectum and colon.

Differently from previous studies, which supposed a possible impact of tumor location and stage on tumor motion itself, we found no correlation between these factors and MM, relying on the better morphological detail provided by MR images [26–28]. Considering these and other differences in the methodology applied in the previous studies, our results are not directly comparable with others.

Our study aimed to assess the factors influencing MM from an MR-based perspective, rather than defining the inter- and intra-fraction movement of GTV during RT treatment. This is a topic of great interest in radiotherapy, especially concerning personalized medicine, dose escalation and organ preservation protocols.

MRgRT is currently considered as one of the best strategies to carry out dose escalation protocols in LARC, in particular with the application of online adaptive (OA) MRgRT protocols, in order to increase the dose-dependent therapeutic efficacy [28–30].

OA MRgRT allows for the re-optimisation of the treatment plan based on the patient’s daily anatomy, improving the precision of treatment delivery in terms of target coverage and OARs sparing, in order to reduce toxicity and maximise treatment opportunities [31, 32].

Furthermore, the application of active gating solutions, which has been shown to be feasible in MRgRT treatment, may allow effective management of intra-fraction movement during nCRT, further reducing the risk of target missing and toxiciy [18].

Our study proposes CTV-PTV margins that can be applied to the mesorectum, basing on a total of 312 MR scans acquired throughout the nCRT treatment of 12 patients, thus reflecting possible treatment induced and other real world modifications in a considerable sample.

Furthermore, the subdivision of the mesorectum into 3 parts allows to apply anisotropic margins to the different sections. Our results suggest that special attention should be paid to the superior and middle sections of the mesorectum, where the anterior portion is characterized by greater mobility.

A limitation of this study is represented by an unequal gender distribution of the patients (male 83.3%, female 16.7%) and thus the differences related to pelvic anatomy (presence of uterus/prostate), the amount and disposition of fat, which were not taken into account in the analysis [33].

In particular, the change in position and shape of the uterus can be very extensive and unpredictable from day to day, therefore affecting the CTV-PTV margins [24].

An interesting possible development of this study is the analysis of the inter- and intra-fraction motion of the GTV, not doable using standard CBCT images, which may be a factor of great interest in the perspective of dose escalation protocols and investigation of OA applications.

## Conclusion

Our MRI study shows that the margins suggested to take into account the overall MM are 1.5 cm in the right-left direction, 1.3 cm posteriorly, 1 cm caudally, 0.5 cm cranially, and that the widest displacement occurs in the anterior direction, whereby 2 cm of margin should be considered.

We have also reported the differences in the MM in its three sections, superior, middle and lower, along with the margins to be considered for each section in the perspective of a reduction of treatment margins.

## Data Availability

The datasets used analysed during the current study are available from the corresponding author on reasonable request.

## Abbreviations

3D-CRT: 3D conformational radiotherapy
CBCT: Cone beam computed tomography
CR: Clinical response
CT: Computed tomography
CTV: Clinical target volume
ECOG: Eastern cooperative oncology group
EPIDs: Electronic portal imaging devices
FB: Free breathing
GTV: Gross tumor volume
IGRT: Image guided radiotherapy
IMA: Inferior mesenteric artery
IMRT: Intensity modulated radiation therapy
LARC: Locally advanced rectal cancer
LE: Local excision
Mlow: Lower mesorectum
MM: Mesorectum motion
Mmid: Middle mesorectum
MR: Magnetic resonance
MRgRT: Magnetic resonance guided radiotherapy
Msup: Superior mesorectum
nCRT: Neoadjuvant chemoradiotherapy
OA: Online adaptive
OARs: Organs at risk
PCC: Pearson correlation coefficient
pCR: Pathological complete response
PTV: Planning target volume
RT: Radiotherapy
SA: Sacral artery
SIB2: Simultaneous integrated boost 2
SRA: Superior rectal artery
TME: Total mesorectal excision
TRUFI: True fast imaging

## References

[1] Arbea L, Ramos LI, Martínez-Monge R, Moreno M, Aristu J (2010). Intensity-modulated radiation therapy (IMRT) vs. 3D conformal radiotherapy (3DCRT) in locally advanced rectal cancer (LARC): dosimetric comparison and clinical implications. Radiat Oncol.

[2] Burbach JPM, den Harder AM, Intven M, van Vulpen M, Verkooijen HM, Reerink O (2014). Impact of radiotherapy boost on pathological complete response in patients with locally advanced rectal cancer: a systematic review and meta-analysis. Radiother Oncol.

[3] Gwynne S, Webster R, Adams R, Mukherjee S, Coles B, Staffurth J (2012). Image-guided radiotherapy for rectal cancer: a systematic review. Clin Oncol (R Coll Radiol).

[4] Valentini V, Gambacorta MA, Barbaro B, Chiloiro G, Coco C, Das P (2016). International consensus guidelines on clinical target volume delineation in rectal cancer. Radiother Oncol.

[5] Ippolito E, Mertens I, Haustermans K, Gambacorta MA, Pasini D, Valentini V (2008). IGRT in rectal cancer. Acta Oncol.

[6] Rosa C, Caravatta L, Di Tommaso M, Fasciolo D, Gasparini L, Di Guglielmo FC (2021). Cone-beam computed tomography for organ motion evaluation in locally advanced rectal cancer patients. Radiol Med.

[7] Daly ME, Murphy JD, Mok E, Christman-Skieller C, Koong AC, Chang DT (2011). Rectal and bladder deformation and displacement during preoperative radiotherapy for rectal cancer: are current margin guidelines adequate for conformal therapy?. Pract Radiat Oncol.

[8] Alickikus ZA, Kuru A, Aydin B, Akcay D, Gorken IB (2020). The importance of mesorectum motion in determining PTV margins in rectal cancer patients treated with neoadjuvant radiotherapy. J Radiat Res.

[9] Chong I, Hawkins M, Hansen V, Thomas K, McNair H, O’Neill B (2011). Quantification of organ motion during chemoradiotherapy of rectal cancer using cone-beam computed tomography. Int J Radiation Oncol Biol Phys.

[10] Yamashita H, Takenaka R, Sakumi A, Haga A, Otomo K, Nakagawa K (2015). Analysis of motion of the rectum during preoperative intensity modulated radiation therapy for rectal cancer using cone-beam computed tomography. Radiat Oncol.

[11] Nuyttens JJ, Robertson JM, Yan D, Martinez A (2002). The variability of the clinical target volume for rectal cancer due to internal organ motion during adjuvant treatment. Int J Radiat Oncol Biol Phys.

[12] Allen SD, Gada V, Blunt DM (2007). Variation of mesorectal volume with abdominal fat volume in patients with rectal carcinoma: assessment with MRI. Br J Radiol.

[13] Peltrini R, Sacco M, Luglio G, Bucci L (2020). Local excision following chemoradiotherapy in T2-T3 rectal cancer: current status and critical appraisal. Updates Surg.

[14] Dossa F, Chesney TR, Acuna SA, Baxter NN (2017). A watch-and-wait approach for locally advanced rectal cancer after a clinical complete response following neoadjuvant chemoradiation: a systematic review and meta-analysis. Lancet Gastroenterol Hepatol.

[15] Corradini S, Alongi F, Andratschke N, Belka C, Boldrini L, Cellini F (2019). MR-guidance in clinical reality: current treatment challenges and future perspectives. Radiat Oncol.

[16] Meldolesi E, Chiloiro G, Giannini R, Menghi R, Persiani R, Corvari B (2022). The role of simultaneous integrated boost in locally advanced rectal cancer patients with positive lateral pelvic lymph nodes. Cancers (Basel).

[17] Rödel C, Graeven U, Fietkau R, Hohenberger W, Hothorn T, Arnold D (2015). Oxaliplatin added to fluorouracil-based preoperative chemoradiotherapy and postoperative chemotherapy of locally advanced rectal cancer (the german CAO/ARO/AIO-04 study): final results of the multicentre, open-label, randomised, phase 3 trial. Lancet Oncol.

[18] Chiloiro G, Boldrini L, Meldolesi E, Re A, Cellini F, Cusumano D (2019). MR-guided radiotherapy in rectal cancer: first clinical experience of an innovative technology. Clin Transl Radiat Oncol.

[19] Glynne-Jones R, Wyrwicz L, Tiret E, Brown G, Rödel C, Cervantes A (2017). Rectal cancer: ESMO clinical practice guidelines for diagnosis, treatment and follow-up†. Ann Oncol Elsevier.

[20] Ng M, Leong T, Chander S, Chu J, Kneebone A, Carroll S (2012). Australasian gastrointestinal trials Group (AGITG) contouring atlas and planning guidelines for intensity-modulated radiotherapy in anal cancer. Int J Radiat Oncol Biol Phys.

[21] Quirke P, Steele R, Monson J, Grieve R, Khanna S, Couture J (2009). Effect of the plane of surgery achieved on local recurrence in patients with operable rectal cancer: a prospective study using data from the MRC CR07 and NCIC-CTG CO16 randomised clinical trial. Lancet.

[22] Nijkamp J, de Jong R, Sonke J-J, Remeijer P, van Vliet C, Marijnen C (2009). Target volume shape variation during hypo-fractionated preoperative irradiation of rectal cancer patients. Radiother Oncol.

[23] Kusters M, Wallner C, Lange MM, DeRuiter MC, van de Velde CJH, Moriya Y (2010). Origin of presacral local recurrence after rectal cancer treatment. Br J Surg.

[24] Tournel K, De Ridder M, Engels B, Bijdekerke P, Fierens Y, Duchateau M (2008). Assessment of intrafractional movement and internal motion in radiotherapy of rectal cancer using megavoltage computed tomography. Int J Radiat Oncol Biol Phys.

[25] Brierley JD, Dawson LA, Sampson E, Bayley A, Scott S, Moseley JL (2011). Rectal motion in patients receiving preoperative radiotherapy for carcinoma of the rectum. Int J Radiat Oncol Biol Phys.

[26] Burbach JPM, Verkooijen HM, Intven M, Kleijnen J-PJE, Bosman ME, Raaymakers BW (2015). RandomizEd controlled trial for pre-operative dose-escalation BOOST in locally advanced rectal cancer (RECTAL BOOST study): study protocol for a randomized controlled trial. Trials.

[27] Kleijnen J-PJE, van Asselen B, Van den Begin R, Intven M, Burbach JPM, Reerink O (2019). MRI-based tumor inter-fraction motion statistics for rectal cancer boost radiotherapy. Acta Oncol.

[28] Eijkelenkamp H, Boekhoff MR, Verweij ME, Peters FP, Meijer GJ, Intven MPW (2021). Planning target volume margin assessment for online adaptive MR-guided dose-escalation in rectal cancer on a 1.5 T MR-Linac. Radiother Oncol.

[29] Chiloiro G, Cusumano D, Boldrini L, Romano A, Placidi L, Nardini M (2022). THUNDER 2: THeragnostic Utilities for neoplastic DisEases of the rectum by MRI guided radiotherapy. BMC Cancer.

[30] Gani C, Boldrini L, Valentini V (2019). Online MR guided radiotherapy for rectal cancer. New opportunities. Clin Transl Radiat Oncol.

[31] Intven MPW, de Mol van Otterloo SR, Mook S, Doornaert PAH, de Groot-van Breugel EN, Sikkes GG, et al. Online adaptive MR-guided radiotherapy for rectal cancer; feasibility of the workflow on a 1.5T MR-linac: clinical implementation and initial experience. Radiother Oncol. 2021;154:172–8. 10.1016/j.radonc.2020.09.024 32976875

[32] Boldrini L, Intven M, Bassetti M, Valentini V, Gani C (2021). MR-Guided radiotherapy for rectal cancer: current perspective on organ preservation. Front Oncol.

[33] Torkzad MR, Blomqvist L (2005). The mesorectum: morphometric assessment with magnetic resonance imaging. Eur Radiol.

